# Is There a Vicious Cycle Between Parental Burnout and Parent–Adolescent Conflict? A Three‐Wave Within‐Family Analytic Approach

**DOI:** 10.1111/famp.70015

**Published:** 2025-03-03

**Authors:** Beiming Yang, Zexi Zhou, Yang Qu, Bin‐Bin Chen

**Affiliations:** ^1^ School of Education and Social Policy Northwestern University Evanston Illinois USA; ^2^ Department of Human Development and Family Sciences The University of Texas at Austin Austin Texas USA; ^3^ Department of Psychology Fudan University Shanghai China

**Keywords:** family conflict, family dynamics, parent–adolescent conflict, parental burnout

## Abstract

Parental burnout is a chronic condition of experiencing exhaustion, inefficacy, and emotional distance in one's parental role. Given the detrimental influence of parental burnout on both parents and children, it is important to study the antecedents and consequences of parental burnout, particularly at the within‐family level. Using a three‐wave sample of 443 Chinese parents (70% mothers; mean age = 41.81 years, SD = 3.81 years) of middle school adolescents (50% girls; mean age = 13.35 years, SD = 0.36 years), the present study examined the transactional processes between parental burnout and parent–adolescent conflict. Random intercept cross‐lagged panel modeling allowed the present study to focus on within‐family effects by using random intercepts to account for between‐family effects. In this way, this study can rule out time‐invariant confounds by focusing on whether the ups and downs of parental burnout at a family level contribute to the changes in parent–adolescent conflict, and vice versa. At the within‐family level, parental burnout predicted greater parent–adolescent conflict over time, and parent–adolescent conflict also predicted greater parental burnout over time. Notably, multigroup comparisons showed that the link from parent–adolescent conflict to parental burnout was only significant among parents with lower but not higher educational attainment, and the link from parental burnout to parent–adolescent conflict was only evident among mothers but not fathers. Taken together, the findings suggest that parental burnout and parent–adolescent conflict positively shape and sustain one another over time, highlighting the necessity to adapt the designs of family conflict interventions in treating and preventing parental burnout.

Parenting can be stressful. Parents under enduring and overwhelming parenting stress may experience parental burnout, which is a chronic condition of feeling exhausted by one's parental role and emotionally distanced from one's children (Mikolajczak et al. 2019). Parental burnout has recently begun to receive increasing scholarly attention, with a rising number of studies focusing on both its antecedents and consequences (for a review, see Mikolajczak et al. 2023). For example, past studies have examined a wide range of etiological factors of parental burnout, including demographics, parents' stable traits, parenting practices, and family functioning (Mikolajczak and Roskam 2018; Mikolajczak et al. 2019). Moreover, longitudinal studies have documented the detrimental effects of parental burnout on adolescents' psychological and behavioral development (Chen et al. 2022; Yang et al. 2021). However, less is known about the relation between parental burnout and parent–child relationship, particularly at a within‐family level. Parent–adolescent conflict may be particularly relevant to parental burnout because of its associations with parents' mental health issues (Burke 2003; Kuhlman et al. 2016). Given the importance of exploring longitudinal changes at the within‐person level (Berry and Willoughby 2017), the present study employed a three‐wave longitudinal design with Chinese parents and adolescents to investigate the bidirectional relations between parental burnout and parent–adolescent conflict on both the between‐family level and the within‐family level. Moreover, multigroup analyses were conducted to examine whether such bidirectional relations differ across families with different demographic backgrounds.

Adolescence is a time during which children increasingly seek individuation from parents, which can be stressful for both parents and adolescents (Koepke and Denissen 2012). Therefore, it is crucial to study the dynamics between parental burnout and parent–adolescent conflict during this developmental period. There may be bidirectional relations between parental burnout and parent–adolescent conflict. First, parental burnout may contribute to greater parent–adolescent conflict over time. Prior cross‐sectional and longitudinal studies have consistently demonstrated that parenting stress is a risk factor for worse parent–adolescent relationships such as dampened parent–adolescent closeness, parent–adolescent communication, and attachment security (Chung et al. 2020; Deater‐Deckard 2008; Ponnet et al. 2013). When parents suffer from parental burnout, they may be too exhausted to meet their children's needs (Mikolajczak et al. 2019). Moreover, parents with parental burnout tend to engage in more negative parenting practices such as parental neglect and hostility toward adolescents (Brianda et al. 2020; Chen et al. 2022), and such punishment‐oriented parenting practices may induce greater conflict between parents and adolescents. Second, parent–adolescent conflict may also contribute to greater parental burnout over time. Family conflict has long been considered a significant stressor for both parents and adolescents (Kuhlman et al. 2016; Repetti et al. 2011). Greater parent–adolescent conflict may exact a toll on parents' health (McEwen 1998), leaving them with less energy to engage in interactions with adolescents (Sun et al. 2021). Indeed, past longitudinal research suggests that parent–adolescent conflict predicts increased parenting stress and parents' psychological stress over time (Cherry et al. 2019; Kim et al. 2023). Under increased stress, parents may question their ability to play the parental role. In this case, heightened conflict with children may put parents over their limit, contributing to parents' feelings of exhaustion and inefficiency, which are some defining characteristics of parental burnout (Mikolajczak et al. 2019). Taken together, parental burnout and parent–adolescent conflict may reciprocally shape one another and develop into a vicious cycle.

Several past studies have used cross‐lagged models to study the bidirectional relations between parents' mental health and other family factors. For example, research on depression found positive bidirectional relations between parental depression and a variety of adolescent developmental problems (e.g., internalizing and externalizing problems; Lowthian et al. 2023; Van Steijn et al. 2014), such that parental depression contributes to greater maladjustment among adolescents and vice versa. Research on parental burnout also suggests that there are positive bidirectional relations between parental burnout and negative parenting practices (Mikolajczak et al. 2019). Specifically, parental burnout predicts parents' greater use of neglect and violence, and such negative parenting practices further contribute to greater parental burnout (Mikolajczak et al. 2019). However, no prior research—to the best of our knowledge—has focused on the changes in parental burnout at the within‐family level. Scholars have called attention to the study of longitudinal changes at the within‐person rather than the between‐person level (Berry and Willoughby 2017). From a theoretical perspective, within‐person models are more aligned with developmental theory because of their focus on developmental changes (Curran and Bauer 2011; Hoffman and Stawski 2009). From a methodological perspective, by examining how the intraindividual ups and downs of one variable relate to another, within‐person models are better at ruling out time‐invariant confounding factors such as genetics (Hamaker et al. 2015). Relatedly, as within‐person models are resilient to time‐invariant confounders, findings at the within‐person level can aid causal inference (Rohrer and Murayama 2023), making it valuable for potential interventions. Overall, given the great interest in how parental burnout is developed and how parental burnout can be treated, it is important to explore the antecedents and consequences of parental burnout while disentangling the within‐family variance from the between‐family variance.

It is largely unclear whether the dynamics between parental burnout and parent–adolescent conflict would vary across parents with different demographics (e.g., parents' gender, adolescents' gender, and parents' educational attainment). Past studies on parental burnout found that mothers reported slightly higher parental burnout than fathers (Mikolajczak et al. 2018; Roskam et al. 2018). Meanwhile, fathers (vs. mothers) may be more vulnerable to parental burnout when they experience an imbalance of risks over resources in parental demands (Roskam and Mikolajczak 2020). Very few studies on parental burnout included adolescents' gender as a variable. Limited evidence suggested that parents of adolescent boys may be at a higher risk of parental burnout than parents of adolescent girls (Wang et al. 2021). Regarding educational attainment, although it is typically associated with a greater ability to cope with stress (Christensen et al. 2006; Cooper et al. 2009; Merritt et al. 2004), the findings on the association between parents' educational attainment and parental burnout are quite mixed (Mikolajczak et al. 2018; Séjourné et al. 2018; Sodi et al. 2020). On the one hand, parents with higher educational attainment may have more resources to balance out parenting stress. On the other hand, parents with higher educational attainment may have more difficulties in balancing work and parenting duties, as they typically experience greater job demands (Solomon et al. 2022). Overall, given these demographic differences related to parental burnout, it is possible that the potential bidirectional links between parental burnout and parent–adolescent conflict would differ between (1) mothers versus fathers, (2) parents of girls versus parents of boys, and (3) parents with high versus low levels of educational attainment. Nevertheless, no study to date has examined whether these demographic characteristics would moderate either the effect of parental burnout on family environment or the effect of family environment on parental burnout. Therefore, according to the current state of the literature, it remains unclear which direction these differences may occur.

## 1 The Present Study

Using three‐wave longitudinal data from Chinese parents of middle school children, the current study examined the transactional processes between parental burnout and parent–adolescent conflict with random intercepts cross‐lagged panel model (RI‐CLPM; Hamaker et al. 2015). Compared to traditional CLPM, RI‐CLPM includes additional latent random intercepts that account for between‐person effects, which allows the model to examine within‐person effects with the cross‐lagged paths (Hamaker et al. 2015). We hypothesized that parental burnout and parent–adolescent conflict would be positively associated with one another at the within‐family level. That is, within a family, parental burnout would contribute to greater parent–adolescent conflict over time, and parent–adolescent conflict would contribute to greater burnout over time. In addition to the hypothesized reciprocal relations between parental burnout and parent–adolescent conflict, we also sought to explore whether demographic characteristics would moderate such bidirectional relations. Using multigroup analyses, the current study examined whether the bidirectional relations between parental burnout and parent–adolescent conflict would differ across (1) mothers versus fathers, (2) parents of girls versus parents of boys, and (3) parents with high versus low levels of educational attainment.

## 2 Method

### 2.1 Participants

The sample consisted of 443 Chinese families. Parents were primary caregivers in the family (70% mothers; mean age = 41.81 years, SD = 3.81 years), and their children were seventh‐grade adolescents (50% girls; mean age = 13.35 years, SD = 0.36 years). Participants were recruited from three middle schools in Shanghai. In terms of achievement, one school was above average and the other two were average achieving. With regard to educational attainment, 49% of parents had education beyond high school (e.g., a bachelor's or master's degree), which was similar to the local average in Shanghai (Shanghai Bureau of Statistics 2022).

### 2.2 Procedure

Participants completed the same online questionnaires three times over a year, with 6 months between each wave of data collection. At each wave, extensive explanations of the research were given, and participants completed the online consent before taking the questionnaire. Ethical approval for the study was obtained from the Institutional Review Board of the School of Social Development and Public Policy at Fudan University. Families received small gifts for their participation. Among the full sample of 443 families who participated at Wave 1, a total of 307 families participated at Wave 3. The attrition rate from Wave 1 to Wave 3 was 31%. Among all the families that completed the questionnaires at Wave 1, 83% of them completed follow‐up surveys at either Wave 2 or Wave 3. Results in Little's MCAR test (*χ* ^2^ = 38.64, df = 16, *p* = 0.001) suggested that missing cases were not missing completely at random (MCAR; Little 1988). Compared to participants who completed all three waves of questionnaires, those who had missing data reported higher parent–adolescent conflict at baseline (*F* = 9.20, *p* = 0.004). To handle missing data, full information maximum likelihood estimation was used to provide reliable standard errors under a wide range of conditions (Schafer and Graham 2002; Wothke 2000). A priori power analysis for structural equation modeling was conducted. With a probability level of 0.05, a small to medium effect size (*f* ^2^ = 0.25; Cohen 1988), six observed variables, and eight latent variables, 271 participants were required to achieve a power of 0.80 (Westland 2010). Therefore, the sample size of 443 families is adequate for the study.

### 2.3 Measures

#### 2.3.1 Parental Burnout

At each wave, parental burnout was assessed using the Parental Burnout Assessment (PBA; Roskam et al. 2018). On a 7‐point Likert scale ranging from 0 (never) to 6 (daily), parents reported on the frequency of their feelings of parental burnout such as exhaustion and irritation related to parenting (23 items; e.g., “I feel completely run down by my role as a parent” and “I cannot take being a parent anymore”). At each wave, the mean was taken across all items, with higher numbers indicating greater parental burnout (*α*s = 0.97 at wave 1, 0.97 at wave 2, and 0.98 at wave 3).

#### 2.3.2 Parent–Adolescent Conflict

At each wave, parent–adolescent conflict was assessed using the Parent‐Adolescent Conflict Scale (PACS; Ruiz and Gonzales 1998). On a 5‐point Likert scale ranging from 1 (almost never) to 5 (almost always), parents reported on the frequency of conflict between the adolescent and the parent (10 items, e.g., “you and your child had a serious argument or fight” and “you and your child yelled or raised voices at each other”). At each wave, the mean was taken across all items, with higher numbers indicating greater parent–adolescent conflict (*α*s = 0.93 at wave 1, 0.94 at wave 2, and 0.95 at wave 3).

#### 2.3.3 Demographic Characteristics

Demographic characteristics, including adolescents' age, adolescents' gender, parents' age, parents' gender, and parents' educational attainment were assessed in the present study. Adolescents' gender was coded as 0 = boy and 1 = girl. Parents' gender was coded into 0 = man and 1 = woman. Parents' educational attainment was coded as 0 = less than a college degree and 1 = college degree or higher.

### 2.4 Overview of the Analyses

**FIGURE 1:**
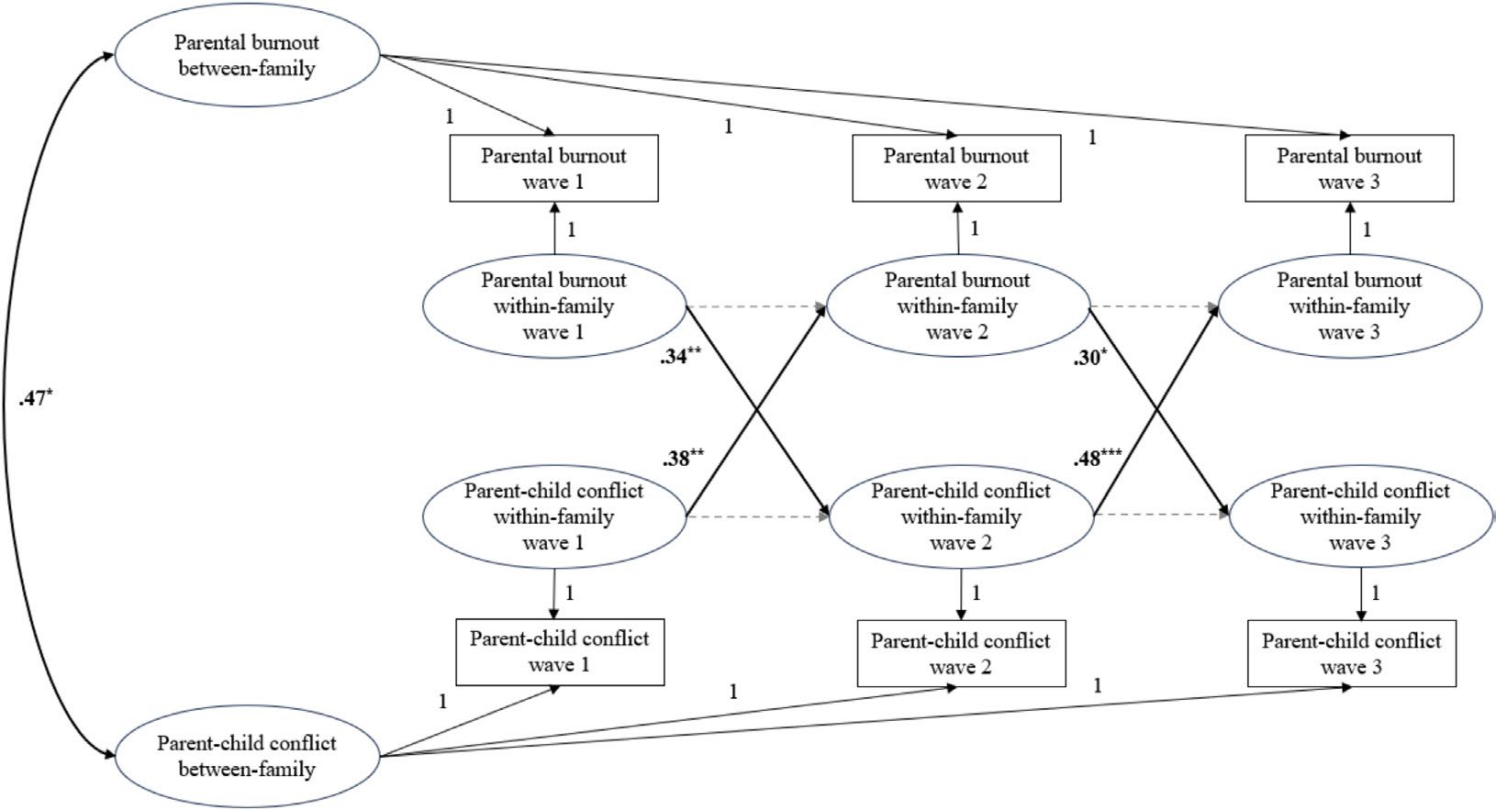
Random intercept cross‐lagged panel model for parental burnout and parent–adolescent conflict. For the ease of presentation, concurrent correlations at the within‐family level are not pictured (significant between parental burnout and parent–adolescent conflict at each wave) but can be found in Table 2. Significant paths and standardized estimates are shown in bold. The faded and dashed arrows indicate paths that were estimated but were not statistically significant. *p < 0.05. **p < 0.01. ***p < 0.001.

To test the hypothesized bidirectional relations between parental burnout and parent–adolescent conflict, RI‐CLPM in the context of structural equation modeling (SEM) was conducted using MPlus 8.9 (Muthén and Muthén 2022). RI‐CLPM disentangles the within‐person effects from the between‐person effects by including both autoregressive paths and random intercepts (Hamaker et al. 2015). As shown in Figure 1, between‐family effects were estimated by the correlation between the random intercepts of parental burnout and parent–adolescent conflict, whereas the within‐family effects were estimated by the cross‐lagged paths between within‐family variables of parental burnout and parent–adolescent conflict. Following the recommended practices of RI‐CLPM (Hamaker et al. 2015; Muthén and Muthén 2022), loadings of random intercepts and within‐family variables were constrained to one, and measurement error variances were constrained to zero. In addition, correlations between within‐family variables at wave 1 and between residuals of within‐family variables at wave 2 and wave 3 were also included in the model.

Multigroup analyses were conducted to examine whether the bidirectional relations between parental burnout and parent–adolescent conflict differ between (1) fathers versus mothers, (2) parents of adolescent boys versus parents of adolescent girls, and (3) parents with lower versus higher educational attainment. Following the practices of past studies (e.g., Lozada et al. 2021; Thompson et al. 2020; Van Heel et al. 2020), in order to reduce model complexity, parameters were constrained across waves for multigroup comparison. For multigroup RI‐CLPMs, models with cross‐lagged parameters unconstrained between groups and models with cross‐lagged parameters constrained between groups were conducted. To examine whether the bidirectional paths differ between groups, chi‐square difference tests were used to compare unconstrained models with constrained models.

Supplementary analyses were conducted to rule out potential demographic confounds. With the inclusion of random intercepts in the RI‐CLPM, the within‐person effects are assumed to be unaffected by time‐invariant covariates (Allison 2009; Hamaker et al. 2015). Nevertheless, a model with demographic covariates added was estimated as supplementary analyses. Bivariate correlations between demographic variables (i.e., adolescents' age, gender, parents' age, gender, and educational attainment) and the key variables (i.e., parental burnout and parent–adolescent conflict) were examined. Demographic variables showing significant correlations with key variables at specific time points were then included as covariates in the model. Demographic variables with nonsignificant correlations were not included to avoid overcontrol and decreased statistical power (Becker 2005).

## 3 Results

### 3.1 Descriptive Statistics and Bivariate Correlations

Table 1 shows the descriptive statistics and correlations of the variables. Parental burnout was associated with greater parent–adolescent conflict at each wave (*r*s > 0.45, *p*s < 0.001) as well as across all waves (*r*s > 0.19, *p*s < 0.001). Parents of older adolescents reported higher burnout at wave 1, but not at wave 2 or 3. Parents with higher educational attainment reported lower burnout at waves 2 and 3, but not at wave 1. Adolescents' gender, parents' gender, and parents' age were not related to burnout or conflict at any wave.

**TABLE 1:** Descriptive statistics and correlations of variables.

	1	2	3	4	5	6	7	8	9	10	11
Wave 1											
1. Parental burnout	—										
2. Parent–adolescent conflict	0.45***	—									
Wave 2											
3. Parental burnout	0.54***	0.40***	—								
4. Parent–adolescent conflict	0.40***	0.42***	0.58***	—							
Wave 3											
5. Parental burnout	0.48***	0.27***	0.54***	0.43***	—						
6. Parent–adolescent conflict	0.19***	0.36***	0.30***	0.46***	0.50***	—					
Demographic variables											
7. Adolescent age	0.11*	−0.04	−0.01	0.04	0.05	−0.02	—				
8. Parent age	0.04	−0.03	−0.07	0.00	−0.07	0.05	0.11	—			
9. Adolescent gender	−0.05	−0.02	−0.04	−0.01	−0.01	0.00	−0.11	0.00	—		
10. Parent gender	0.02	−0.09	0.02	0.05	0.04	−0.02	−0.10	−0.29***	−0.00	—	
11. Parent education	−0.05	−0.09	−0.12*	−0.10	−0.13*	−0.09	−0.02	0.12*	−0.03	0.04	—
Mean	0.66	2.00	0.58	1.92	0.66	1.94	13.35	41.81	0.49	0.70	0.49
SD	0.91	0.73	0.81	0.73	0.89	0.77	0.36	3.81	—	—	—
Min	0	1	0	1	0	1	12.27	32	0	0	0
Max	6	5	6	5	5.09	5	15.45	57	1	1	1

*Note:* For adolescents' gender, 0 = boy and 1 = girl. For parents' gender, 0 = man and 1 = woman. For parental education, 0 = less than a college degree, and 1 = college degree or higher.

*p < 0.05 and ***p < 0.001.

### 3.2 Bidirectional Relations Between Parental Burnout and Parent–Adolescent Conflict

RI‐CLPM in the context of SEM was conducted to examine the bidirectional relations between parental burnout and parent–adolescent conflict. The model fit was excellent, CFI = 1.00, TLI = 1.00, RMSEA = 0.02, and SRMR = 0.01. Based on intra‐class correlations, 53% of the variance in parental burnout and 41% of the variance in family conflict were explained by between‐family differences, with the rest explained by within‐family fluctuations. The findings are presented in Figure 1, and more details are shown in Table 2. At the between‐family level, parental burnout was associated with greater parent–adolescent conflict (*β* = 0.47, *p* = 0.01). At the within‐family level, all four cross‐lagged paths were significant. Specifically, parental burnout at wave 1 predicted greater parent–adolescent conflict at wave 2 (*β* = 0.34, *p* = 0.002); parental burnout at wave 2 predicted greater parent–adolescent conflict at wave 3 (*β* = 0.30, *p* = 0.03); parent–adolescent conflict at wave 1 predicted greater parental burnout at wave 2 (*β* = 0.38, *p* = 0.002); parent–adolescent conflict at wave 2 predicted greater parental burnout at wave 3 (*β* = 0.48, *p* < 0.001). Moreover, at the within‐family level, none of the autoregressive paths was significant (*β*s < 0.13, *p*s > 0.31), and all the concurrent associations between burnout and conflict were significant (*β*s > 0.45, *p*s < 0.007).

**TABLE 2:** RI‐CLPM linking parental burnout, parent–adolescent conflict, and attachment security.

	*β*	SE	*t*	*p*
Cross‐lagged effects				
Burnout (1) → conflict (2)	**0.34**	0.11	3.04	0.002
Burnout (2) → conflict (3)	**0.30**	0.14	2.15	0.032
Conflict (1) → burnout (2)	**0.38**	0.12	3.03	0.002
Conflict (2) → burnout (3)	**0.48**	0.12	3.89	< 0.001
Autoregressive paths				
Burnout (1) → burnout (2)	0.07	0.13	0.51	0.611
Burnout (2) → burnout (3)	0.03	0.15	0.21	0.837
Conflict (1) → conflict (2)	0.09	0.13	0.65	0.516
Conflict (2) → conflict (3)	0.13	0.13	1.01	0.311
Between‐family associations				
Burnout with conflict	**0.47**	0.18	2.54	0.011
Within‐family concurrent associations				
Burnout (1) with conflict (1)	**0.45**	0.10	4.58	0.007
Burnout (2) with conflict (2)	**0.66**	0.08	8.83	< 0.001
Burnout (3) with conflict (3)	**0.48**	0.06	7.74	< 0.001

*Note:* Significant coefficients are highlighted in bold. (1) = wave 1, (2) = wave 2, (3) = wave 3. Burnout = parental burnout. Conflict = parent–adolescent conflict.

### 3.3 Multigroup Analyses

Three sets of multigroup RI‐CLPMs were conducted to examine whether the bidirectional relations between parental burnout and parent–adolescent conflict differ across parents' educational attainment, adolescents' gender, and parents' gender. First, fathers were compared to mothers. The multigroup RI‐CLPM showed excellent model fit, CFI = 1.00, RMSEA = 0.00, SRMR = 0.03. The chi‐square difference test showed that the results differed between these two groups (*χ* ^2^ = 6.04, *p* = 0.05). Parent–adolescent conflict predicted greater parental burnout in both groups (fathers: *β* = 0.74, *p* < 0.001; mothers: *β* = 0.22, *p* = 0.01). However, parental burnout predicted greater parent–adolescent conflict only among mothers (*β* = 0.42, *p* < 0.001), but not among fathers (*β* = 0.26, *p* = 0.15).

Second, parents of girls were compared to parents of boys. The multigroup RI‐CLPM showed good model fit, CFI = 0.99, RMSEA = 0.05, and SRMR = 0.04. The chi‐square difference test showed that the results did not differ between these two groups (*χ* ^2^ = 2.77, *p* = 0.25). In both groups, parental burnout predicted greater parent–adolescent conflict (boys: *β* = 0.45, *p* < 0.001; girls: *β* = 0.28, *p* = 0.04) and parent–adolescent conflict predicted greater parental burnout (boys: *β* = 0.29, *p* = 0.004; girls: *β* = 0.55, *p* < 0.001).

Finally, parents with less than a college degree were compared to parents with a college degree or higher. The multigroup RI‐CLPM showed acceptable model fit, CFI = 0.98, RMSEA = 0.07, and SRMR = 0.08. The chi‐square difference test showed that the results differed between these two groups (*χ* ^2^ = 17.88, *p* < 0.001). Parental burnout predicted greater parent–adolescent conflict in both groups (low education group: *β* = 0.38, *p* = 0.004; high education group: *β* = 0.27, *p* = 0.03). However, parent–adolescent conflict predicted greater parental burnout only among parents with lower educational attainment (*β* = 0.61, *p* < 0.001), but not among parents with higher educational attainment (*β* = −0.03, *p* = 0.84).

### 3.4 Supplementary Analyses

Supplementary analyses including demographic covariates were conducted to rule out potential demographic confounds. As shown in Table 1, among the demographic variables included in this study, adolescents' age was significantly correlated with parental burnout at wave 1, and parents' educational attainment was significantly correlated with parental burnout at wave 2 and wave 3. Therefore, covariances between these demographic variables and parental burnout at specific time points were added to the model. There was no meaningful change to the results after including parents' educational attainment. More specifically, the paths from parental burnout to parent–adolescent conflict (wave 1 to wave 2: *β* = 0.34, *p* = 0.002; wave 2 to wave 3: *β* = 0.29, *p* = 0.04) and the paths from parent–adolescent conflict to parental burnout (wave 1 to wave 2: *β* = 0.38, *p* = 0.003; wave 2 to wave 3: *β* = 0.47, *p* < 0.001) all remained significant.

## 4 Discussion

Parental burnout has attracted increased scholarly attention in recent years. However, little do we know about how parental burnout relates to parent–child relationship. Moreover, no study to date has examined parental burnout at the within‐family level. The current study examined the bidirectional relations between parental burnout and parent–adolescent conflict at a within‐family level. The results of RI‐CLPM suggest that there are reciprocal relations between parental burnout and parent–adolescent conflict at the within‐family level, such that parental burnout and parent–adolescent conflict positively shape and sustain one another over time. In addition, multigroup analyses showed that the link from parent–adolescent conflict to parental burnout was only evident among parents with lower educational attainment but not among parents with higher educational attainment, and the link from parental burnout to parent–adolescent conflict was only evident among mothers but not fathers.

The use of RI‐CLPM allowed the current study to examine between‐family differences, within‐family concurrent associations, within‐family stability, and within‐family bidirectional effects between parental burnout and parent–adolescent conflict. At the between‐family level, the random intercept of parental burnout was positively associated with the random intercept of parent–adolescent conflict. This suggests that families with greater burnout also tend to have greater parent–adolescent conflict. The finding is consistent with past studies on the positive associations between parental burnout and problems in family functioning (e.g., high interparental conflict and low marital satisfaction; Mikolajczak et al. 2018). Parents in dysfunctional families (vs. well‐functioning families) are likely to encounter more difficulties in regard to parenting (Cherry et al. 2019; Kim et al. 2023), and thus they tend to have greater parental burnout and engage in conflict with their children more frequently. At the within‐family level, the variation of parental burnout was positively associated with the variation of parent–adolescent conflict at each time point. This suggests that, compared to each family's own average level, when parents show higher than normal parental burnout, they are also experiencing higher than normal parent–adolescent conflict at the same time. With regard to longitudinal changes, the autoregressive paths of parental burnout and parent–adolescent conflict were not significant. Although both parental burnout and parent–adolescent conflict were moderately to highly correlated across waves (see Table 1), the variation of parental burnout was not predictive of its variation at a later time, and the variation of parent–adolescent conflict was also not predictive of its variation at a later time. This is in line with the argument that autoregressive parameters are typically small in size and can be closer to 0 in RI‐CLPM (Hamaker et al. 2015; Mulder and Hamaker 2021). The findings suggest that, once the trait‐like between‐family variability is excluded, the variations of parental burnout and parent–adolescent conflict have limited stability over time.

Importantly, at the within‐family level, parental burnout predicted greater parent–adolescent conflict over time, and parent–adolescent conflict also predicted greater parental burnout over time. A potential explanation is that heightened parenting stress from experiencing conflict (Cherry et al. 2019; Kim et al. 2023) may push parents over their limit, putting them at risk of greater parental burnout. In turn, neglect and hostility from burned‐out parents (Brianda et al. 2020; Chen et al., 2021) may lead to additional conflict between parents and children. By demonstrating parent–adolescent conflict as both an antecedent and a consequence of parental burnout, the current study adds important empirical evidence to the understanding of parental burnout. The current study extends the literature on parental burnout as the first to focus on the link between parental burnout and parent–child relationship and also the first to examine the longitudinal changes in parental burnout at the within‐family level. The findings also have crucial practical implications. Whereas interventions on parental burnout are extremely scarce (Mikolajczak et al. 2023), there are plenty of successful interventions on parent–adolescent conflict in various cultural contexts (e.g., Johnson et al. 2005; Kindsvatter and Desmond 2013; Oveisi et al. 2010). Given the longitudinal link between parent–adolescent conflict and parental burnout at the within‐family level, effective interventions for reducing parent–adolescent conflict may also reduce parental burnout over time. Therefore, future interventions may consider adapting the designs of family conflict interventions in treating and preventing parental burnout.

Limited gender differences were found in this study. Neither parents' gender nor adolescents' gender was associated with the mean level of parental burnout and parent–adolescent conflict. With regard to the bidirectional link between parental burnout and parent–adolescent conflict, adolescents' gender did not play a moderating role and parents' gender only moderated one direction of the relations—only mothers' but not fathers' parental burnout was predictive of later parent–adolescent conflict. This finding is somewhat different from a past study with a cross‐sectional design, which suggests that the association between parental burnout and parental neglect is stronger for fathers than for mothers (Roskam and Mikolajczak 2020). Differences in methodologies (e.g., longitudinal vs. cross‐sectional), concepts of the measures (parent–child relationship vs. parenting behavior), and sample characteristics (e.g., age and culture) may contribute to the opposite direction of gender differences. Overall, we still know too little about gender differences in the mechanisms of parental burnout to infer gender‐specific recommendations on intervention and policy. More studies in the future should systematically compare the processes of parental burnout between fathers and mothers and between parents of boys and girls.

Parents' educational attainment moderated the link from parent–adolescent conflict to parental burnout, such that parent–adolescent conflict was only predictive of later parental burnout among parents with low but not high educational attainment. This is in line with a past study showing that the negative impact of chaotic family environments on adolescents was less pronounced among families with high income (Schreier et al. 2014). It has been argued that parents are at risk of parental burnout when risks consistently outweigh resources (Mikolajczak and Roskam 2018). Parents with higher (vs. lower) educational attainment may have more social and financial support to balance out parenting stress, making them more resilient to risk factors of parental burnout, such as parent–adolescent conflict. Such resilience processes among parents with higher educational attainment were also reflected in the direct association between educational attainment and parental burnout. Although parents with higher educational attainment did not experience less parental burnout at baseline, they did experience less burnout at later time points compared to their counterparts with lower educational attainment. In contrast, the path from parental burnout to parent–adolescent conflict did not differ between the low vs. high educational attainment groups. This suggests that once parental burnout has already been formed, its negative consequences can hardly be buffered by additional resources among parents with higher educational attainment.

### 4.1 Limitations and Future Directions

The present study has several limitations that point to directions for future research. First, the current study only examined the moderating role of demographic characteristics, which are time‐invariant factors at the between‐family level. Past research at the between‐family level found that time‐variant variables such as emotion regulation strategies may play a protective role against parental burnout (Yang et al. 2021). Future research should conduct moderation analyses with time‐variant variables at the within‐family level (Speyer et al. 2023). Although this requires a more complicated model and a larger sample to support the model, the findings would be informative because time‐variant factors are potentially easier to intervene. Second, the current research only followed parents of early adolescents over a year. Therefore, it is unclear whether such reciprocal relations between parental burnout and parent–adolescent conflict also apply to parents of younger children. Future studies should consider studying the changes in parental burnout over a longer period of time among parents with younger children. Third, the current study was not able to study the short‐term fluctuations in parental burnout and parent–adolescent conflict. Both parental burnout and parent–adolescent conflict can change on a day‐to‐day basis (Brenning et al. 2023; LoBraico et al. 2020). Therefore, it is important for future research to explore the dynamics between parental burnout and parent–adolescent conflict within a shorter time frame (e.g., on a daily or weekly basis). Finally, the current sample included parents in Shanghai, which has a higher SES than the national average of China. Given that educational attainment played a significant moderating role in the link from parent–adolescent conflict to parental burnout, it is unclear whether the findings can be generalized to parents in other areas with lower SES. Relatedly, it is also necessary for future studies to explore the dynamics between parental burnout and parent–adolescent conflict in other cultures. Social norms of parenting can vary greatly across cultures (Harkness and Super 2002; Lansford 2022). Cross‐cultural research on parental burnout also suggests that the prevalence of parental burnout differs across the globe (Roskam et al. 2021). Therefore, it is key for future studies to study the antecedents and consequences of parental burnout using more diverse samples.

## 5 Conclusions

Enduring exposure to parenting stress puts parents at risk of parental burnout, which has a detrimental influence on both parents and children. The current research is the first to study parental burnout at the within‐family level. The use of RI‐CLPM allowed this three‐wave longitudinal study to rigorously investigate the bidirectional relations between parental burnout and parent–adolescent conflict at the within‐family level by disentangling the between‐family effects. The findings demonstrate parent–adolescent conflict as both an antecedent and a consequence of parental burnout. Moreover, the findings highlight parents with lower educational attainment as a particularly vulnerable group that is at risk of this vicious cycle. Given the close connection between parental burnout and parent–adolescent conflict at the within‐family level, interventions and policy recommendations should consider targeting parent–adolescent conflict to prevent parental burnout.

## Conflicts of Interest

The authors declare no conflicts of interest.
